# Matrix‐Isolation Electron Paramagnetic Resonance Studies of Radical Ions and Neutral Radicals Generated by Radiolysis of Organic Molecules at Cryogenic Temperatures

**DOI:** 10.1002/tcr.202500304

**Published:** 2026-01-06

**Authors:** Kenji Komaguchi

**Affiliations:** ^1^ Applied Chemistry Program Graduate School of Advanced Science and Engineering Hiroshima University Higashi‐Hiroshima Japan

**Keywords:** high‐resolution electron paramagnetic resonance spectroscopy, matrix isolation, radical pair, radicals, second‐order JT effect

## Abstract

Electron paramagnetic resonance (EPR) spectroscopy has long been widely utilized to investigate, characterize, and monitor highly reactive paramagnetic chemical species generated in materials upon exposure to ionizing radiation. This personal account presents EPR observations and spectral analyses of several fundamental paramagnetic species, including cations, anions, and neutral radicals, isolated using a low‐temperature solid matrix isolation (MI) technique combined with radiation exposure, a method in which the author has extensive experience. These findings are not only of significant interest in the field of molecular science but also demonstrate the utility of the MI technique as a laboratory‐based approach to exploring chemical evolution in space. Recent density functional theory analyses, which reveal a second‐order Jahn–Teller distortion, suggest that the stability of the distorted structure of the silacyclohexane radical cation is considerably less pronounced than previously indicated by Hartree–Fock‐based theoretical calculations. Furthermore, EPR results for the perfluorocubane radical anion, a species that has recently attracted significant attention, are also presented.

## Graphical abstract

By selecting an appropriate matrix and irradiating the matrix containing a trace amount of solute at low temperatures, ionic or neutral radicals derived from the solute can be generated and trapped in the matrix. EPR spectroscopy of these samples yields highly detailed information on the radicals, inaccessible by other experimental techniques.

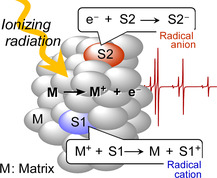

## 1 Introduction

Electron spin paramagnetic resonance (EPR) spectroscopy enables highly sensitive detection of radical species and provides detailed insights into their electronic structure, dynamics, and reactivity. It reveals magnetic interactions between electron and nuclear spins, providing unique molecular‐level insights including their environmental context, which are inaccessible by other spectroscopic techniques [1, 2].

Since the mid‐1950s, many radical ions have been studied using EPR, mainly in liquid phase through redox reactions, electrolysis, or photolysis. Stable radical ions with conjugated systems, like aromatics, were often examined [3]. Subsequently, from the 1960s, radical ions and neutral radicals began to be studied using the matrix isolation (MI) technique employing noble gases as inert matrices [4, 5, 6, 7]. This method enabled the stabilization of highly reactive species at cryogenic temperatures, effectively suppressed intermolecular interactions, and facilitated high‐resolution spectroscopic analysis of their intrinsic electronic properties. Compared to combinations with optical absorption methods such as infrared spectroscopy, the development of noble gas matrix systems for EPR applications has proven to be technically demanding and was limited to only a small number of researchers. Knight and co‐workers advanced the noble‐gas MI‐EPR technique and identified numerous small‐molecule radicals, many of which cannot be generated in halocarbon matrices [8, 9]. In the late 1990s, the author and collaborators carefully prepared argon matrix samples and demonstrated that EPR observation of the methyl radical at 4 K could be achieved with significantly higher resolution than previously reported for similar systems by irradiating the samples with X‐rays [10].

In contrast, the low‐temperature solid‐phase halocarbon MI method was introduced by Shida et al. in 1978 and made it possible to perform steady‐state spectroscopic analysis of *σ*‐type radical cations originating from saturated hydrocarbons, which had previously been difficult [11]. Shida's method employs halogenated carbon compounds with a high ionization potential, such as CFCl_3_, as a matrix. A small amount of solute, serving as a radical cation precursor, is embedded in a solid matrix and irradiated with ionizing radiation [12, 13, 14]. Exploiting ionization potential differences between matrix and solute, the halocarbon MI method selectively generates solute‐derived radical cations for EPR characterization. This simple, effective technique was rapidly adopted for studying *σ*‐type radical cations using various halocarbon matrices [15, 16, 17]. The author conducted EPR measurements using various halocarbon matrices on radical cations of trimethylenemethane, a series of silacycloalkanes, ethylsilane, tetramethylsilane, and fluoroethane [18, 19, 20, 21, 22, 23, 24]. These investigations revealed how partial deuteration influences structural distortion, stability, molecular motion, and reactivity.

In MI techniques utilizing radiation exposure, the formation of radical cations as guest species can be described by reactions (1) and (2) [12, 13, 15, 25]. Upon irradiation, high‐energy photons (radiation) predominantly interact with matrix molecules (M), which are present in excess compared to solute molecules (RH), leading to the generation of primary electrons and matrix cations via radiation‐induced primary chemical reactions such as the photoelectric effect or Compton scattering. These primary electrons possess sufficient energy to further ionize matrix molecules, resulting in secondary ionization (as shown in reaction (1)). The holes formed in the matrix molecules can migrate to solute molecules with lower ionization energies, thereby generating solute radical cations (reaction (2)). Meanwhile, the electrons released in reaction (1) may eventually react with matrix molecules to form stable anion radicals (reaction (3)), or participate in subsequent reactions to produce neutral radicals or more stable anionic species, such as halide anions in the case of halogen‐containing compounds. When the radical cation of the solute is unstable or the sample temperature is increased, it can undergo deprotonation to form a neutral radical (reaction (4)). At higher concentrations, ion–molecule reactions (reaction (5)) can lead to the formation of neutral radicals and dimeric radical cations.


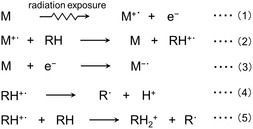


When anionic solute species act as guest molecules, the principle is similar to that for cations, but the matrix must have a lower ionization potential than the solute (i.e., the solute has lower electron affinity). Typical matrices include ethanol, 2‐methylhydrofuran, neopentane, and tetramethylsilane (TMS) [3, 4, 13]. After hole capture, these matrices undergo deprotonation to yield neutral radicals, as in reaction (4). EPR signals from matrix‐derived radicals may overlap with those of the guest species, but this is often mitigated because halogen‐containing anionic radicals generally exhibit broader signals than hydrocarbon radicals [26].

This personal account highlights a series of EPR investigations conducted by the author, utilizing low‐temperature solid‐state MI technique in which radicals are generated via ionizing radiation. High‐resolution EPR observations have revealed several notable radical species. These include methyl radicals and their triplet pairs with hydrogen atoms, as well as H···H_2_ complexes in Ar matrices. In halocarbon matrices, a second‐order Jahn–Teller distortion was identified in silacyclohexane radical cations. In addition, the EPR spectrum of the radical anion of perfluorocubane, recently synthesized for the first time, was successfully measured, providing direct insight into its distinctive electronic structure. These findings, though representing only a small portion of the results obtained by MI‐EPR, these findings highlight the overlooked potential of MI‐EPR in exploring the properties of radical species.

## 2 High‐Resolution EPR Using Argon Matrix

Noble gases are chemically inert, and their solid forms consist of weakly interacting crystals held together by van der Waals forces between spherical atoms [27, 28]. Consequently, they have long been employed as low‐temperature solid matrices for IR and EPR measurements [4]. Among noble gases, Ar is particularly suitable due to its isotopes having zero nuclear spin (*I* = 0), while Ne contains only a negligible fraction (0.27%) of isotopes with nuclear spin (*I* = 2/3) [29]. This minimizes magnetic interactions with guest species, enabling high‐resolution EPR. Moreover, Argon enables measurements over a broader temperature range than neon, particularly below 40 K.

### 2.1 Methyl Radicals

#### 2.1.1 CH_3_ Radical

**FIGURE 1:**
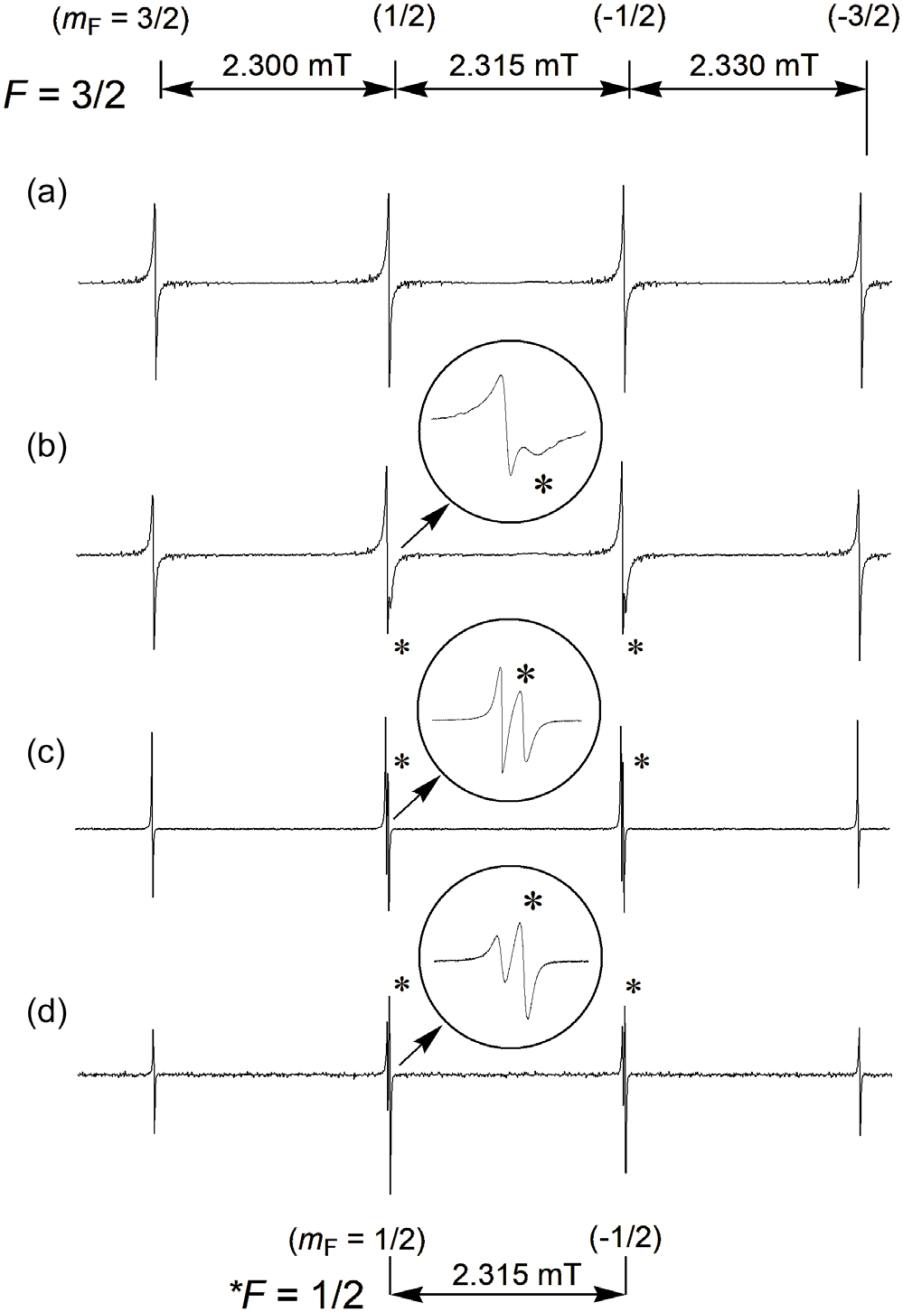
Temperature‐dependent EPR spectra of the CH_3_ radical in an Ar matrix containing 0.2 mol% CH_4_ after X‐ray irradiation at 4.2 K. (a) At 6 K, only the *F* = 3/2 quartet with *A*_1_ symmetry is observed. (b) 12.0 K, (c) 20.0 K, and (d) 40.0 K, where two *F* = 1/2 doublets with *E* symmetry (marked with *) increase in intensity as higher rotational levels become populated. Transitions of *A*_1_ and *E* symmetry are separated by a small second‐order shift in the isotropic part of the hf splitting. Reproduced with permission from ref. [10]. Copyright 1999, American Chemical Society.

Prior to the present study, several EPR studies of CH_3_ radical using noble gas matrices had been published. However, none fully exploited the high‐resolution capabilities of Ar as a matrix. In our experiments, high‐purity Ar containing 0.02–0.1 mol% methane was sealed in a glass vessel (ca. 200 mL, ≈80 kPa), and the tip of the sample tube was cooled to 4 K to condense and freeze the gas mixture. X‐ray irradiation (Cu target; 58 kV, 48 mA) for 30 min enabled successful observation of high‐resolution EPR spectra (Figure 1) [10].

The CH_3_ radical adopts a D_3h_ symmetric structure, in which three hydrogen atoms are bonded via sp^2^ hybrid orbitals of the carbon atom, while the unpaired electron primarily occupies the carbon 2p_z_ orbital. Due to the magnetic interaction between the unpaired electron and the three magnetically equivalent protons, the EPR spectrum splits into four equally spaced lines, with peak intensity ratios following a binomial distribution of 1:3:3:1 [30, 31, 32, 33, 34, 35, 36].

However, a quartet EPR spectrum of equal intensity was observed for the isolated CH_3_ radical below 6 K, exhibiting isotropic hf splitting of 2.315 mT for the proton (^1^H) (after the second‐order hf correction). These spectral features correspond to the A_1_‐lines under D_3_ symmetry [37]. Upon increasing the temperature above 12 K, a new doublet, designated as E‐lines, emerged at the magnetic quantum number positions *m*_F_ = ±1/2, exhibiting the same hf splitting. Notably, the resonance positions of the E‐lines were shifted by 0.024 mT relative to the inner *m*_F_ = ±1/2 components of the A_1_‐lines. This shift is attributed to second‐order hf interactions arising from the difference in total nuclear spin states: *F* = 1/2 (E‐lines) and *F* = 3/2 (A_1_‐lines) (see Section 4.2). The intensity of the E‐lines increased progressively with temperature and exceeded that of the corresponding A_1_‐line components by a factor of two at 40 K. Based on the temperature‐dependent variation in the E/A intensity ratio, the effective rotational energy splitting between the A and E levels in the matrix environment was discussed [37].

**FIGURE 2:**
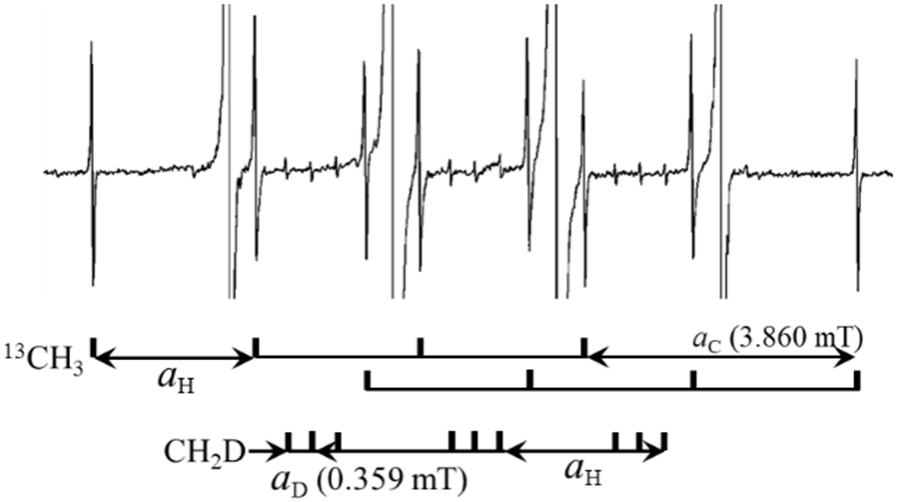
EPR spectrum at 20 K corresponding to Figure 1c, shown with enhanced intensity scale. Multiple isotope satellite peaks are resolved, as indicated by stick diagrams. The prominent double quartet is assigned to the ^13^CH_3_ isotopomer, while the weaker triple triplet corresponds to the CH_2_D radical. Natural abundances of magnetic isotopes are 1.1% for ^13^C and 0.015% for D. Reproduced with permission from ref. [10]. Copyright 1999, American Chemical Society.

Due to the exceptionally high spectral resolution (linewidth of 0.007 mT), EPR signals corresponding to CH_2_D and ^13^CH_3_ radicals were distinctly detected at 20 K, despite their low natural abundances of 0.015% (D) and 1.1% (^13^C), respectively (Figure 2) [29]. The clear observation of such effects in CH_3_ radical, which has a small hf splitting (≈2.3 mT), highlights the high resolution of the measurement. Notably, the EPR spectrum of CH_2_D radical, containing deuterium at its natural abundance (0.015%), was also detected. Since EPR signal intensity is proportional to radical concentration, this high resolution simultaneously implies high sensitivity. While detection of signals from naturally abundant ^13^C (1.1%) in organic radicals is not uncommon, the observation of deuterium signals is particularly remarkable.

Following our studies, Eloranta et al. have conducted detailed EPR observations of CH_3_ radicals in solid noble gas matrices, and solid CO, CO_2_, and N_2_ matrices [38, 39, 40]. Restricted rotation and temperature‐dependent nuclear spin state populations revealed matrix‐specific effects, including anomalous E*/*A ratios and nonrotating species in CO_2_ [40].

#### 2.1.2 CD_3_ Radical

**FIGURE 3:**
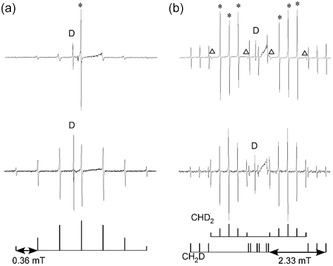
High‐resolution EPR spectra of CD_3_ (a), CH_2_D and CHD_2_ (b) observed in Ar matrices at 4.2 K (top) and 20 K (bottom). Radicals were generated by X‐ray irradiation of Ar matrices containing ≈0.1 mol% of CD4 (a) and of CH_2_D_2_ (b). At 4.2 K, the EPR lineshapes show anomalous hf patterns attributed to quantum effects. For an explanation of the peaks of CHD_2_ marked with * and Δ, refer to the main text. Reproduced with permission from ref. [10]. Copyright 1999, American Chemical Society.

Quantum mechanical effects associated with the perdeuterated methyl radical (CD_3_) were clearly manifested in its EPR lineshape (Figure 3a). At 4.2 K, a pronounced singlet was superimposed on the central peak of the expected septet, which exhibited a hf splitting of 0.359 mT. The observed relative intensity distribution of the septet was 1.0:3.8:7.0:105:8.7:4.3:1.5, deviating significantly from the binomial pattern of 1:3:6:7:6:3:1, primarily due to the anomalously enhanced central transition. This deviation was attributed to the superposition of a “classical” high‐temperature spectrum, resulting from the rapid rotational motion of the CD_3_ radical. The intense central line is indicative of quantum interference effects arising from the bosonic nature of deuterium nuclei (D, nuclear spin quantum number *I* = 1, boson). Notably, the spectral behavior of CD_3_ differs markedly from that of CH_3_, reflecting the influence of nuclear spin statistics and rotational dynamics. With increasing temperature, the intensity of the central line diminished rapidly, and by 10 K, the overall spectral profile converged to the expected binomial distribution. This temperature‐dependent evolution underscores the role of quantum rotational effects in shaping the EPR spectrum of CD_3_.

#### 2.1.3 CH_2_D and CHD_2_ Radicals

Partially deuterated methyl radicals, CH_2_D and CHD_2_, were generated in an Ar matrix by employing CD_2_H_2_ as the precursor molecule (Figure 3b). At 4.2 K, the EPR spectrum of the CHD_2_ radical exhibited a double quintet structure, arising from hf interactions with one ^1^H and two deuterons (D), with respective hf splittings of 2.315 and 0.359 mT. The central triplet component of the quintet (marked with * in Figure 3b) displayed a narrow linewidth of 0.01 mT and a pronounced transition at *m*_F_ = +1, 0, −1, with an intensity ratio of 1:1:1. In contrast, the outer lines at *m*_F_ = ±2 (marked with Δ) exhibited linewidths approximately four times broader. Upon warming to 10 K, the relative peak intensities underwent rapid redistribution, converging to a binomial pattern of 1:2:3:2:1, consistent with a freely rotating CHD_2_ radical. This temperature‐dependent behavior reflects the onset of rotational averaging and quantum statistical effects. In comparison, the 4.2 K spectrum of the CH_2_D radical revealed a triple triplet pattern, attributed to two equivalent protons and one deuteron. The triplet hf lines associated with the two ^1^H nuclei exhibited equal intensity, though not conforming to the expected binomial ratio of 1:2:1. Analogous to the behavior observed for CH_3_, upon warming above 15 K, three additional hf lines emerged on the high‐field side of each central triplet component, shifted by 0.024 mT. The relative intensity of these new lines reached a limiting ratio of 1:1 with the original lines at 40 K (Figure 3), indicating thermal population of rotational states. The relative spectral intensity ratio of [CHD_2_] to [CH_2_D] was ≈8:1 at 4.2 K, significantly deviating from the statistical expectation of 1:1 based on the use of CH_2_D_2_ as the solute. This disparity suggests that the lighter hydrogen atom (^1^H) dissociates more readily from the methane molecule than deuterium, highlighting isotope‐dependent reaction dynamics in the MI process. For details of the lineshape analysis, refer to our previous reports [10, 41].

### 2.2 Radical Pairs of H···CH_3_

Ionizing radiation or photolysis induces the decomposition of singlet molecules into pairs of doublet species (radicals), and the pairwise trapping of such radicals is a fundamental aspect of radiation‐induced damage. When two radicals are confined within a separation of ≈2 nm, they can form either singlet or triplet spin states, with the triplet state being detectable via EPR spectroscopy. Numerous EPR investigations have been conducted on radical pairs generated in irradiated organic crystals, notably including the pioneering study by Kurita on dimethylglyoxime single crystals [42]. However, EPR studies focusing on radical pairs composed of primary organic species, such as the hydrogen atom–methyl radical pair (H···CH_3_), remain relatively scarce. Gordy et al. were the first to report an EPR spectrum attributable to the H···CH_3_ radical pair, separated by a single methane molecule in irradiated solid methane at 4.2 K [43]. The inter‐radical distance of 0.68 nm was deduced from the axially symmetric electron–electron dipolar coupling tensor observed in the spectrum. Further insights into the spatial distribution of H···CH_3_ (and its deuterated analog, D···CD_3_) pairs in irradiated solid CH_4_ (CD_4_) at 4.2 K were provided by Toriyama et al. [44]. Here, highly resolved EPR spectra of radical pairs embedded in irradiated solid Ar matrices are presented, offering novel insights into the structure and dynamics of these fundamental species [41].

**FIGURE 4:**
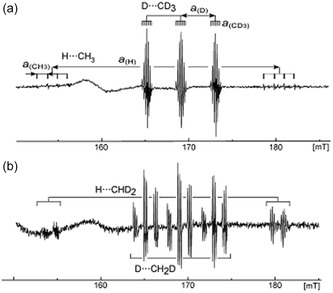
EPR spectra (Δ*m*_s_ = ±2) of hydrogen atom–methyl radical pairs trapped in a solid Ar matrix at 4.2 K. (a) Spectra of H···CH_3_ and D···CD_3_ pairs; (b) Spectra of H···CHD_2_ and D···CH_2_D pairs. Radical pairs were generated via X‐ray irradiation of solid Ar matrices doped with CH_4_–CD_4_ (0.25 mol% each) and CH_2_D_2_ (0.5 mol%), respectively. A broad singlet centered around 160 mT is attributed to unidentified triplet species. Reproduced with permission from ref. [41]. Copyright 2007, American Chemical Society.

As described in the preceding section, high‐resolution EPR spectra of a series of deuterium‐labeled methyl radicals were obtained from X‐ray irradiated solid Ar matrices with 0.5 mol% of various deuterated methane isotopologues, including CH_4_, CH_2_D_2_, and CD_4_, at 4.2 K. Upon increasing the microwave power and modulation amplitude during spectral acquisition, distinct EPR signals corresponding to triplet radical pairs, such as H···CH_3_, H···CHD_2_, D···CH_2_D, and D···CD_3_, were clearly resolved. These signals were observed at both the allowed (Δ*m*_s_ = ±1) and forbidden (Δ*m*_s_ = ±2) transition bands, in addition to the characteristic spectra of isolated ^1^H (or D) atoms and methyl radicals (CH_3_, CH_2_D, CHD_2_, and CD_3_). High‐resolution EPR spectra of H···CH_3_ radical pairs were examined in detail. At the forbidden Δ*m*_s_ = ±2 transition, three distinct sets of doublets with splittings in the range of 25.1–26.3 mT were observed, each further resolved into quartets with a hf splitting of 1.16 mT. These double‐quartet structures correspond closely to one‐half of the isotropic hf splittings of isolated hydrogen atoms (*a*_iso_ = 51.8 mT) and CH_3_ radicals (*a*_iso_ = 2.3 mT), indicating that the observed signals originate from the triplet state of the H···CH_3_ radical pair. This triplet state lies energetically below the singlet state by 2*J* (the singlet‐triplet separation), where |*J*| >> *a*_iso_(isotropic hf splitting of H atom) ≈1.4 GHz. At the allowed Δ*m*_s_ = ±1 transition, the ^1^H hf lines, split by one‐half of *a*_iso_(H) and *a*_iso_(CH_3_), exhibited further anisotropic splitting into doublets due to the parallel (*d*_*||*_) and perpendicular (*d*) components of the electron–electron dipolar coupling (i.e., zero‐field splitting). Three distinct sets of fine structure parameters, corresponding to *d* values of 4.2, 4.9, and 5.1 mT, were clearly resolved. The formation of radical pairs was further substantiated by the observation of analogous spectra for D···CD_3_ pairs in irradiated CD_4_/Ar matrices, and H···CHD_2_ and D···CH_2_D pairs in irradiated CH_2_D_2_/Ar systems (Figure 4) [41]. Notably, only two distinct radical pairs, H···CH_3_ and D···CD_3_, were detected in the equimolar CH_4_–CD_4_ mixture (Figure 4a), indicating that the hydrogen atom and methyl radical within each pair originate from the same methane molecule.

**FIGURE 5:**
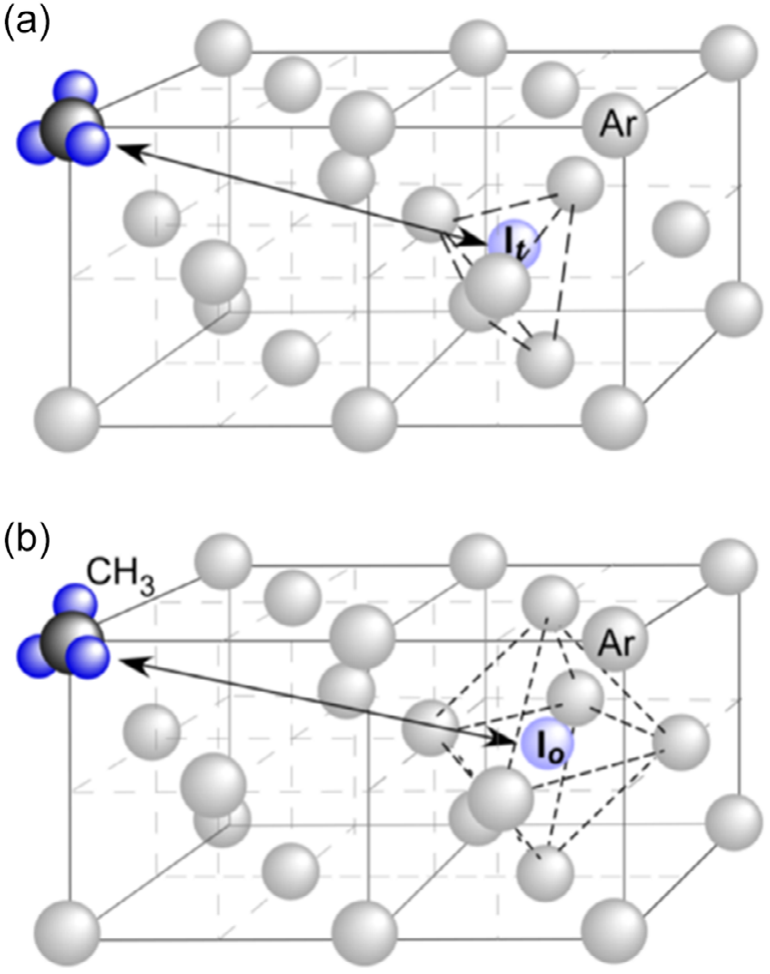
Schematic representation of the proposed trapping sites for the H···CH_3_ radical pair in solid Ar, based on a dipolar coupling value of *d* = 4.2 mT (corresponding to *R* = 0.87 nm). The CH_3_ radical is assumed to occupy a substitutional site in the face‐centered cubic (fcc) lattice of solid Ar. The associated hydrogen atom is proposed to reside either (a) at an interstitial tetrahedral site (I_t_), located 0.87 nm from the CH_3_ radical, or (b) at an interstitial octahedral site (I_o_), located 0.88 nm away, or potentially at both sites depending on local lattice conditions. Reproduced with permission from ref. [41]. Copyright 2007, American Chemical Society.

The separation distance *R* of the H···CH_3_ radical pair was estimated using the point–dipole interaction model, expressed as *R* = (3*gβ*/2*d*)^1/3^ where *g* is the *g*‐value of the radical, *β* is the Bohr magneton, and *d* is the experimentally observed perpendicular component of the dipolar coupling tensor, *d* [1, 2]. Substituting the observed values of *d* = 4.2, 4.9, and 5.1 mT yields separation distances of *R* = 0.87, 0.83, and 0.82 nm, respectively. In solid Ar matrices, three distinct trapping sites are known: the interstitial tetrahedral site (I_t_), the interstitial octahedral site (I_o_), and the substitutional site within the face‐centered cubic (fcc) lattice [41]. Assuming the CH_3_ radical occupies a substitutional site, consistent with the location of its parent methane molecule, the most probable position of the paired H atom is either the I_t_ site at 0.87 nm or the I_o_ site at 0.88 nm from the CH_3_ radical. These distances align closely with the experimentally derived values, particularly the 0.87 nm separation, as shown in Figure 5. Further support for the proposed trapping site of the H atom comes from its observed hf splitting of 51.8 mT, which is notably larger than the gas‐phase value of 50.8 mT. This enhancement is consistent with previous findings that hydrogen atoms confined in narrower environments, such as the interstitial tetrahedral site, exhibit increased ^1^H hf splittings [27, 45, 46, 47].

Finally, it is noteworthy that radical pairs such as H···NH_2_ and D···ND_2_ have been detected by EPR spectroscopy in solid Ar and krypton matrices at 4 K [48]. In related work, Knight and collaborators have reported EPR studies on triplet‐state radical pairs involving deuterium‐substituted hydrogen atoms, namely H···H, H···D, and D···D, generated and stabilized in noble gas matrices at 4.2 K. These findings were interpreted using a theoretical framework that treats the spin pairs as weakly interacting atomic species [49, 50]. Furthermore, triplet radical pairs of isotopically substituted nitrogen atoms, including ^14^N···^14^N, ^14^N···^15^N, and ^15^N···^15^N, have also been reported by Knight et al. [51].

### 2.3 Hydrogen Atom…Hydrogen Molecule Complex

As discussed in the preceding section, solid Ar is well suited for high‐resolution EPR spectroscopy due to its chemically inert nature and the absence of nuclear hf interactions (*I* = 0 for all stable isotopes). Its face‐centered cubic (fcc) structure has a nearest‐neighbor distance of 0.376 nm, closely matching that of solid hydrogen (hcp, 0.379 nm), suggesting that H_2_ molecules can be uniformly incorporated into the Ar lattice at substitutional sites.

#### 2.3.1 Hyperfine Splittings of H Atoms in Solid Argon

**FIGURE 6:**
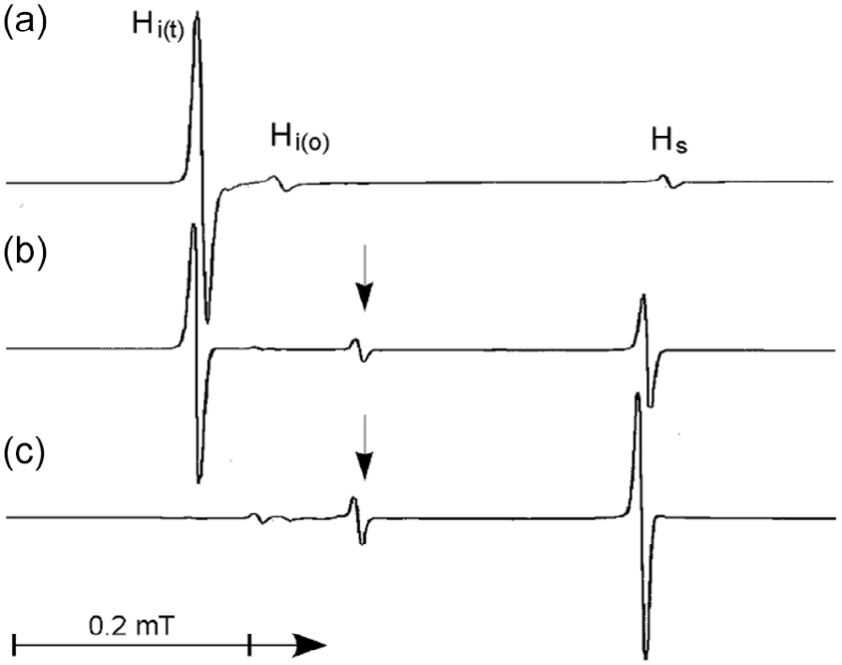
Temperature dependence of the low‐field components of the EPR spectra for hydrogen atoms in a solid Ar matrix containing ≈1.0 mol% H_2_. (a) Spectrum observed at 4.2 K immediately after X‐ray radiolysis at the same temperature. (b) Spectrum obtained observed at 13 K after annealing the sample from 4.2 K. (c) Spectrum observed at 20 K. Reproduced with permission from ref. [45]. Copyright 1997, Elsevier.

**FIGURE 7:**
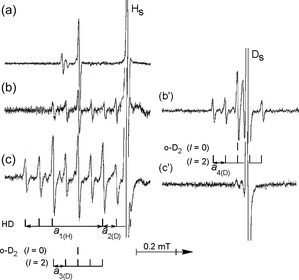
Low‐field components of the EPR spectra for hydrogen atoms (a, b, and c) and deuterium atoms (b' and c') in solid Ar matrices containing: (a) 1.0 mol% H_2_, (b) and (b') 1.0 mol% D_2_, and (c) and (c') 3.3 mol% HD, all recorded at 20 K. The H and D atoms were generated by X‐ray radiolysis of the samples at 4.2 K. Reproduced with permission from ref. [45]. Copyright 1997, Elsevier.

Figure 6 shows low‐field components of the EPR spectra of H atoms generated by X‐ray radiolysis of a solid Ar matrix containing ≈1.0 mol% H_2_ molecules at 4.2 K. Three distinct doublet signals corresponding to hf splittings of ^1^H at 51.54, 51.42, and 50.73 mT are observed [45]. These values exceed the theoretical gas‐phase hf splitting of 50.8 mT, indicating that the hydrogen atoms are confined within narrower lattice sites in the solid Ar matrix. This increase in hf splitting is due to the Pauli exclusion effect, which becomes more effective than van der Waals interactions in narrower trapping sites in the solid Ar matrix. Based on these observations, the H atoms are assigned to three distinct lattice sites: the interstitial tetrahedral site (H_i(t)_), the interstitial octahedral site (H_i(o)_), and the substitutional site (H_s_). Immediately after the radiolysis at 4.2 K (Figure 6a), the doublet at of 51.54 mT exhibits the highest intensity, suggesting that over 90% of the generated hydrogen atoms preferentially occupy the narrowest site, H_i(t)_. Upon annealing the sample to 13 K, H atoms initially trapped at H_i(t)_ were found to migrate to H_s_, accompanied by a decrease in the total radical concentration due to recombination via the reaction H + H → H_2_. This migration process was completed below 20 K, and the most intense peak is attributed to H atoms occupying H_s_. Concurrently, a new doublet signal with a ^1^H hf splitting of 51.18 mT, indicated by the arrow in Figure 6, appeared at 13 K. Figure 7 shows the low‐field components of the EPR spectra observed at 20 K, corresponding to newly formed H and D atoms in solid Ar matrices containing trace amounts of H_2_, D_2_, and HD. In the Ar matrix containing HD (Figure 7c), the doublet signal further resolved into nine isotropic lines due to super‐hf interactions, with splittings of 0.068 and 0.062 mT for the outer and inner three lines, respectively. The relative peak intensity followed a pattern of 1:1:2:1:2:1:2:1:1. This behavior contrasts sharply with the spectra obtained from the Ar matrix containing H_2_, where no super‐hf splitting was observed (Figure 7a). In the case of deuterium atoms generated in irradiated Ar matrices containing D_2_, five isotropic super‐hf lines with a splitting of 0.062 mT and a relative intensity of 1:1:2:1:1 were detected (Figure 7b'). However, no super‐hf features were observed for newly formed deuterium atoms in the Ar matrix containing HD (Figure 7c').

#### 2.3.2 Formation of Complex via Tunneling Reaction

During the migration of H atoms in a solid Ar matrix containing HD, both H and D atoms may independently encounter HD molecules occupying H_s_. The interaction pathways are governed by two competing reactions:

(6) $$\mathrm{HD}+D\;\to \;H+D_{2}\;\left(\text{exothermic}\right)$$

(7) $$\mathrm{HD}+H\;\to \;D+H_{2}\;\left(\text{endothermic}\right)$$

As reaction (6) is energetically favorable, D atoms are expected to undergo tunneling‐assisted conversion with HD, yielding H atoms that subsequently become trapped near D_2_ molecules. In contrast, due to the endothermic nature of reaction (7), H atoms are more likely to be immobilized in the vicinity of HD molecules without undergoing conversion to D atoms.

The super‐hf structure observed in the EPR spectrum of H atoms in the HD containing Ar matrix, characterized by nine isotropic lines, can be interpreted as a superposition of two distinct complexes: H···D_2_ (reaction product) and H···HD (unreacted precursor). The spectrum attributed to the H···HD complex is expected to exhibit a double triplet pattern, arising from super‐hf splittings with both the H and D nuclei. As illustrated by the stick diagrams in Figure 7c, the hf splitting for hydrogen, *a*_1_(H), is theoretically predicted to be 6.514 times greater than that for deuterium, *a*_2_(D).

Focusing now on the H···D_2_ complex, it is essential to consider both *para*‐D_2_ (*p*‐D_2_) and *ortho*‐D_2_ (*o*‐D_2_) as possible partner molecules forming the complex with the H atom. Due to the bosonic nature of deuterons (nuclear spin *I* = 1), *p*‐D_2_ and *o*‐D_2_ occupy rotational states with quantum numbers *J* = odd and *J* = even, respectively. In the ground state, *o*‐D_2_ (*J* = 0) is energetically favored over *p*‐D_2_ (*J* = 1) by ≈85 K [52]. Under normal conditions, conversion between *o*‐D_2_ and *p*‐D_2_ is quantum mechanically forbidden in the absence of magnetic interactions. However, the presence of nearby H or D atoms can act as magnetic perturbations, facilitating the conversion of adjacent *p*‐D_2_ molecules into *o*‐D_2_ in the lowest rotational state (*J* = 0) at cryogenic temperatures. This conversion enables the formation of *o*‐D_2_ with total nuclear spin quantum numbers *I* = 0 and *I* = 2. Consequently, the EPR spectrum of the H···D_2_ complex exhibits five isotropic super‐hf lines with relative peak intensities of 1:1:2:1:1, corresponding to the hf splitting, *a*_3_(D). The super‐hf structure arises from a singlet contribution due to *o*‐D_2_ (*I* = 0) and five equally intense lines from *o*‐D_2_ (*I* = 2), reflecting the allowed spin states in the complex.

This interpretation is supported by the experimental observation that no super‐hf structure is detected for the H···H_2_ complex. This absence implies that the *o*‐H_2_ (*I* = 1) molecule paired with the H atom undergoes conversion into *p*‐H_2_ (*I* = 0), which does not contribute to the super‐hf structure. Furthermore, the same super‐hf pattern observed for the H···D_2_ complex is also evident in the D···D_2_ complex, as shown in Figure 7b', reinforcing the interpretation.

Finally, it is worth noting that the spin density of the unpaired electron on each of the three hydrogen atoms in the complex has been theoretically calculated as a function of the distance between the hydrogen atom and the H_2_ molecule [45]. By comparing the experimentally observed super‐hf splittings with the theoretical values, the average distance between the H atom and the H_2_ molecule was estimated to be 0.173 nm. In the temperature range of 24.4–29.3 K, the complex undergoes thermal decay via an activation process. The potential energy barrier for this process was experimentally determined to be 3.5 kJ mol^−1^ in solid Ar, which is approximately half the value reported for hydrogen atoms (6–7 kJ mol^−1^) trapped in octahedral interstitial sites in solid Ar at 16.2–17.2 K [53].

## 3 Structural Distortion of Silacyclohexane Radical Cations

Shiotani et al. first reported that radical cations of methylcyclohexane (Me‐*c*C_6_) and 1,1‐dimethylcyclohexane (1,1‐Me_2_‐*c*C_6_) adopt asymmetrically distorted structures with C_1_ symmetry, characterized by elongation of one adjacent C—C bond [54, 55, 56]. Since then, many radical cations with similar distortions have been identified, including various cycloalkane, silacycloalkane, diethylsilane, and their derivatives [19, 20, 21, 22, 57, 58, 59]. Experimental and theoretical studies show that one‐electron oxidation intrinsically lowers molecular symmetry in alkane radical cations, even in systems without Jahn–Teller activity, provided the parent molecule has C_s_, C_2_, or C_2v_ symmetry.

Silacyclohexane (*c*SiC_5_) is a stable saturated six‐membered ring compound with C_s_ symmetry in geometrical structure, featuring two equivalent Si—C bonds. Since the ionization potential of silicon is higher than that of carbon, the unpaired electron in the radical cation is expected to be primarily localized on the Si—C bonds. Therefore, the presence or absence of structural distortion upon one‐electron oxidation can be assessed by examining whether the two Si—C bonds remain equivalent. Moreover, unlike linear molecules, the cyclic structure eliminates concerns about gauche‐type conformations. For these reasons, *c*SiC_5_ radical cation was selected as the target of this study.

### 3.1 EPR Spectra of Silacyclohexane Radical Cations

The EPR spectra of the radical cations of *c*SiC_5_ and 1‐methylsilacyclohexane (1‐Me‐*c*SiC_5_) at 4.2 K in *c*C_6_F_12_ and CF_3_‐*c*C_6_F_11_ matrices were successfully analyzed using the ^1^H hf splittings given in Table 1, employing spectral simulation techniques. The 4.2 K spectrum of *c*SiC_5_−2,2,6,6‐*d*_4_^+^ exhibits a doublet of doublets with hf splittings of 7.55 and 2.85 mT, each corresponding to a single ^1^H. Similarly, the spectrum of 1‐Me‐*c*SiC_5_−2,2‐*d*_2_^*+*^ displays a double doublet with splittings of 3.0 and 2.0 mT, in addition to those observed for 1‐Me‐*c*SiC_5_−2,2,6,6‐*d*_4_^+^ [21]. These spectral features are interpreted under the assumption of a statically asymmetric *C*_1_ distorted structure for *c*SiC_5_^+^, wherein the unpaired electron is predominantly localized on one of the two Si—C bonds, specifically the Si—C(2) bond. This localization implies that the Si—C bond bearing higher spin density is weakened and elongated.

**TABLE 1:** Experimental hf splittings (in mT) and their assignments for radical cations of silacyclohexane (*c*SiC_5_) and 1‐methyl‐silacyclohexane (1‐Me‐*c*SiC_5_) in a CF_3_‐*c*C_6_F_11_ matrix at 4.2 K [21].

Radical cation^a^	Equatorial at C(2)^b^	Equatorial at C(3)	Equatorial at C(5)	Equatorial at C(6)
*c*SiC_5_^+^	2.60	7.55	2.85	3.45
*c*SiC_5_−2,2,6,6‐*d*_4_^+^		7.55	2.85	
1‐Me‐*c*SiC_5_^+^	2.0	7.3	2.4	3.0
1‐Me*‐c*SiC_5_−2,2,6,6‐*d*_4_^+^		7.3	2.4	

a See Figure 8 for the numbering.

b “Equatorial at C(2)” refers to the equatorial hydrogen atom bonded to the carbon atom C(2) in the radical cation. The same notation applies to other cases.

Based on this structural model, the observed hf splittings of 7.3 and 2.4 mT for 1‐Me‐*c*SiC_5_^+^ are reasonably assigned to the equatorial protons H_3e_ and H_5e_, respectively. The additional splittings of 2.0 and 3.0 mT are attributed to the equatorial protons at C(2) and C(6), namely H_2e_ and H_6e_. The proposed C_1_ asymmetric structure is further corroborated by density functional theory (DFT) calculations, as illustrated in Figure 8.

**FIGURE 8:**
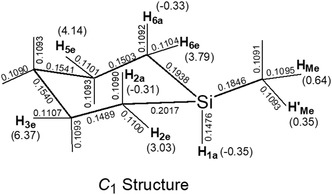
Optimized *C*_1_ geometry of 1‐Me‐*c*SiC_5_^+^ obtained via DFT calculations at the B3LYP/6‐311+G(2df, p) level using Gaussian 16W [60]. The bond lengths are given in nm. Values in parentheses represent the isotropic ^1^H hf splittings (in mT), evaluated for the optimized structure.

DFT calculations at the B3LYP/6‐311+G(2df, p) level show that the energy difference between the asymmetric distorted structure and the C_s_‐symmetric structure of the 1‐Me‐*c*SiC_5_ radical cation is only 23.4 J mol^−1^, indicating nearly equal stability of the two structures. The Si—C bond length difference (0.008 nm) is significantly smaller than that obtained from UHF calculations (0.025 nm) in our previous study [21], suggesting that the UHF method overestimates distortion. The DFT‐calculated isotropic hf splittings for the two hydrogen atoms with high spin density (H_3e_ and H_5e_) are more balanced and closer to experimental values, indicating a more accurate description of the electronic structure.

### 3.2 Origin of the Structural Distortion

The observed structural distortion can be rationalized within the framework of thesecond‐order Jahn–Teller theory, also referred to as the pseudo Jahn–Teller effect [61, 62]. According to this theory, vibronic coupling between the ground and electronically excited states, whose strength is inversely proportional to the energy gap between these states, can lead to adistortion of molecular geometry. Based on this theory, the energy of a radical cation in its ground electronic state can be expressed as follows:

(8) $$E=E_{0}+Q\left\langle \psi_{0}\left|\frac{\partial U}{\partial Q}\right|\psi_{0}\right\rangle +\frac{Q^{2}}{2}\left\langle \psi_{0}\left|\frac{\partial^{2}U}{\partial Q^{2}}\right|\psi_{0}\right\rangle \sum_{k}\frac{{\left[Q\left\langle \psi_{0}\left|\frac{\partial U}{\partial Q}\right|\psi_{k}\right\rangle \right]}^{2}}{\left(E_{0}-E_{k}\right)}$$

*E*_0_ is the energy of the undistorted structure, *Q* is the displacement along a normal coordinate, and *U* is the potential energy from nuclear–nuclear and nuclear–electronic interactions. *ψ*_0_ and *ψ*_k_ are the wavefunctions of the ground and excited electronic states, respectively. For asymmetric displacements, the linear term vanishes due to its odd nature. The third (positive) and fourth (negative) terms determine the preference for distortion. If their sum is positive, the symmetric structure is favored; if negative, the asymmetric structure is preferred. Importantly, the magnitude of the fourth term is inversely related to the energy separation between the ground and low‐lying excited electronic states. In cases where this energy difference is less than a few electronvolts, as observed for the present radical cations, the destabilizing fourth term dominates, thereby stabilizing the asymmetrically distorted C_1_ structure over the symmetric one.

### 3.3 Temperature Dependence of EPR Spectra

**FIGURE 9:**
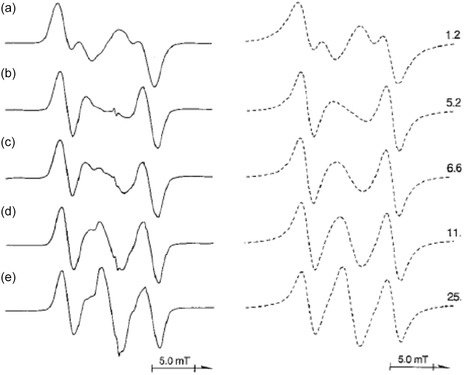
Temperature‐dependent EPR spectra of 1‐Me‐*c*SiC_5_−2,2,6,6‐*d*_4_^+^ observed in a CF_3_‐*c*C_6_F_11_ matrix: (a) 4.2 K, (b) 77 K, (c) 110 K, (d) 130 K, and (e) 140 K. The dotted lines in the right column represent simulated spectra, with the exchange rate constant *k* (in 10^7^ s^−1^) used in the calculations indicated. Reproduced with permission from ref. [21]. Copyright 1997, American Chemical Society.

**FIGURE 10:**
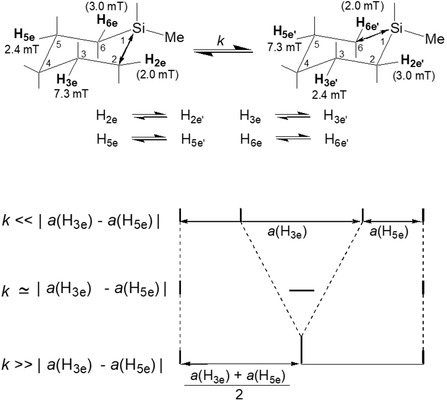
Schematic diagram illustrating the dependence of the EPR lineshape of 1‐Me‐*c*SiC_5_^+^ on the intramolecular hydrogen exchange rate *k* (s^−1^) between two asymmetrically distorted *C*_1_ structures. Reproduced with permission from ref. [21]. Copyright 1997, American Chemical Society.

As an illustrative example, the temperature‐dependent EPR spectra of 1‐Me‐*c*SiC_5_−2,2,6,6‐*d*_4_^+^ were examined (Figure 9). At 4.2 K, the spectrum exhibits a doublet of doublets with ^1^H hf splittings of 7.3 and 2.4 mT, where the inner doublet is less intense than the outer one. Upon increasing the temperature, a new singlet signal emerges at the central field position, with its intensity progressively increasing. At 140 K, the spectrum evolves into a triplet with a splitting of 4.9 mT and a relative intensity distribution approximating a binomial pattern. This spectral transformation isattributed to a dynamic intramolecular exchange between two H atoms, H_3e_ and H_5e_. The 1‐Me‐*c*SiC_5_ radical cation possesses two energetically equivalent mirror‐image structures: one in which the Si—C(2) bond is elongated, and the other in which the Si—C(6)bond is elongated (Figure 8). Figure 10 schematically illustrates how the observed hf splittings of 7.3 and 2.4 mT are averaged through this exchange process,characterized by a rate constant *k* (s^−1^).

The weaker inner doublet observed in the 4.2 K EPR spectrum of 1‐Me‐*c*SiC_5_−2,2,6,6‐*d*_4_^*+*^ can be interpreted by assuming partial averaging of the isotropic hf splittings of H_3e_ and H_5e_ due to an intramolecular exchange process. This process occurs with a rate constant *k* (s^−1^) slightly smaller than the difference in hf splittings between the two protons, i.e., |*a*(H_3e_) − *a*(H_5e_)| ≈ 1.3 × 10^7^ s^−1^. When *k* approaches this value, the inner doublet is expected to vanish, as experimentally observed around 40 K. Upon further temperature increase, a new singlet signal emerges at the central field position, with its intensity growing steadily, indicating a continued increase in the exchange rate. Arrhenius plots of the rate constants *k*(s^−1^) for all silacyclohexane radical cations studied reveal a nonlinear temperature dependence over the range of 4.2 to 130 K. Assuming a linear relationship in the higher temperature region (60–130 K), an activation energy of ≈1.3 kJ mol^−1^ was estimated for both *c*SiC_5_^+^ and 1‐Me‐*c*SiC_5_^+^ in the CF_3_‐*c*C_6_F_11_ matrix. Interestingly, below 40 K, the rate constants were almost independent of temperature with values of *k* = 3.6 × 10^7^ s^−1^ for *c*SiC_5_^+^ and *k* = 1.2 × 10^7^ s^−1^ for 1‐Me‐*c*SiC_5_^+^. These findings suggest that the exchange process is thermally activated at higher temperatures but may proceed via quantum tunneling or other low‐temperature mechanisms in the cryogenic regime.

Structural distortions in allyl‐type radicals have been linked to thesecond‐order Jahn–Teller effect [61, 62, 63]. Similardistortions were also observed in *σ*‐type radical cations of n‐propane, n‐pentane, their methyl‐substitutedderivatives with C_s_ symmetry, and norbornane, with matrix environments stabilizing the deformations, especially in more polar matrices [16, 64, 65, 66, 67]. MO calculations on the norbornane radical cation further support matrix‐induced distortion [66]. In contrast, nearly identical structural distortions of *c*SiC_5_^+^ and 1‐Me‐*c*SiC_5_^+^ are observed across various matrices, suggesting that the distortion originates from its intrinsic electronic structure, with minimal matrix influence.

## 4 Perfluorocubane Radical Anions

**FIGURE 11:**
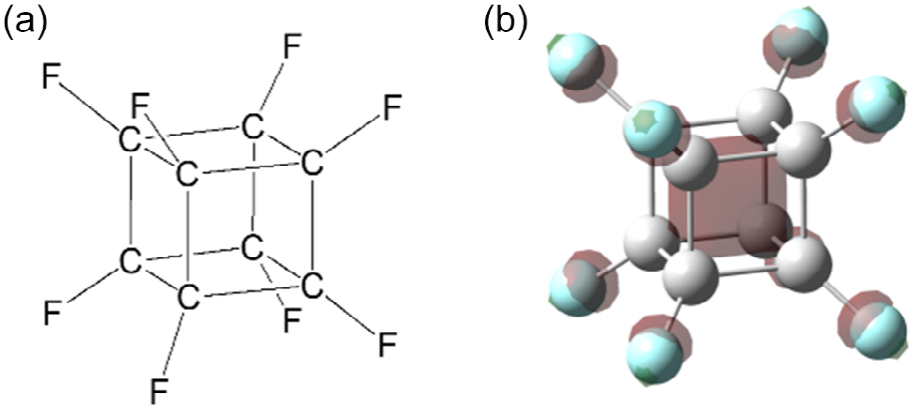
(a) Schematic representation of a C_8_F_8_ molecule (*O*_h_ symmetry); (b) LUMO (*a*_1g_, isovalue = 0.06) of C_8_F_8_ calculated at the B3LYP/6‐311++G(d, p) level [60, 68].

The unprecedented synthesis of perfluorocubane (C_8_F_8_, Figure 11a), in which all cubane hydrogen atoms are replaced by fluorines, marked a major breakthrough in 2022, generating significant impact and widespread attention in the scientific community [69]. The remarkable attention is attributed not merely to its elegant cubic carbon framework, but more importantly to its distinctive electronic configuration.

### 4.1 Predicted Electronic Structure

A theoretical study conducted in 2004 demonstrated that the substitution of eight fluorine atoms significantly lowers the energy level of the lowest unoccupied molecular orbital (LUMO), resulting in a relatively large positive electron affinity of approximately +1.6 eV. Furthermore, a particularly interesting feature of this anion is that the extra electron is confined within the cubane framework.

The LUMO of C_8_F_8_ is primarily composed of C—F antibondingorbitals. Due to the high electronegativity of fluorine, the σ* antibonding orbital of the C—F bond is dominated by contributions from the carbon 2p orbitals rather than those of fluorine. These carbon 2p orbitals overlap in‐phase along the C—F bond axes within the cubane framework. Consequently, an additional electron can be effectively confined within the cubane cage, contributing to the stabilization of the perfluorocubane radical anion. For reference, the LUMO of C_8_F_8_ obtained from DFT calculations using the B3LYP/6‐311++G(d, p) method is shown in Figure 11b [60, 68, 69].

### 4.2 EPR Spectra of C_8_F_8_ Radical Anion

**FIGURE 12:**
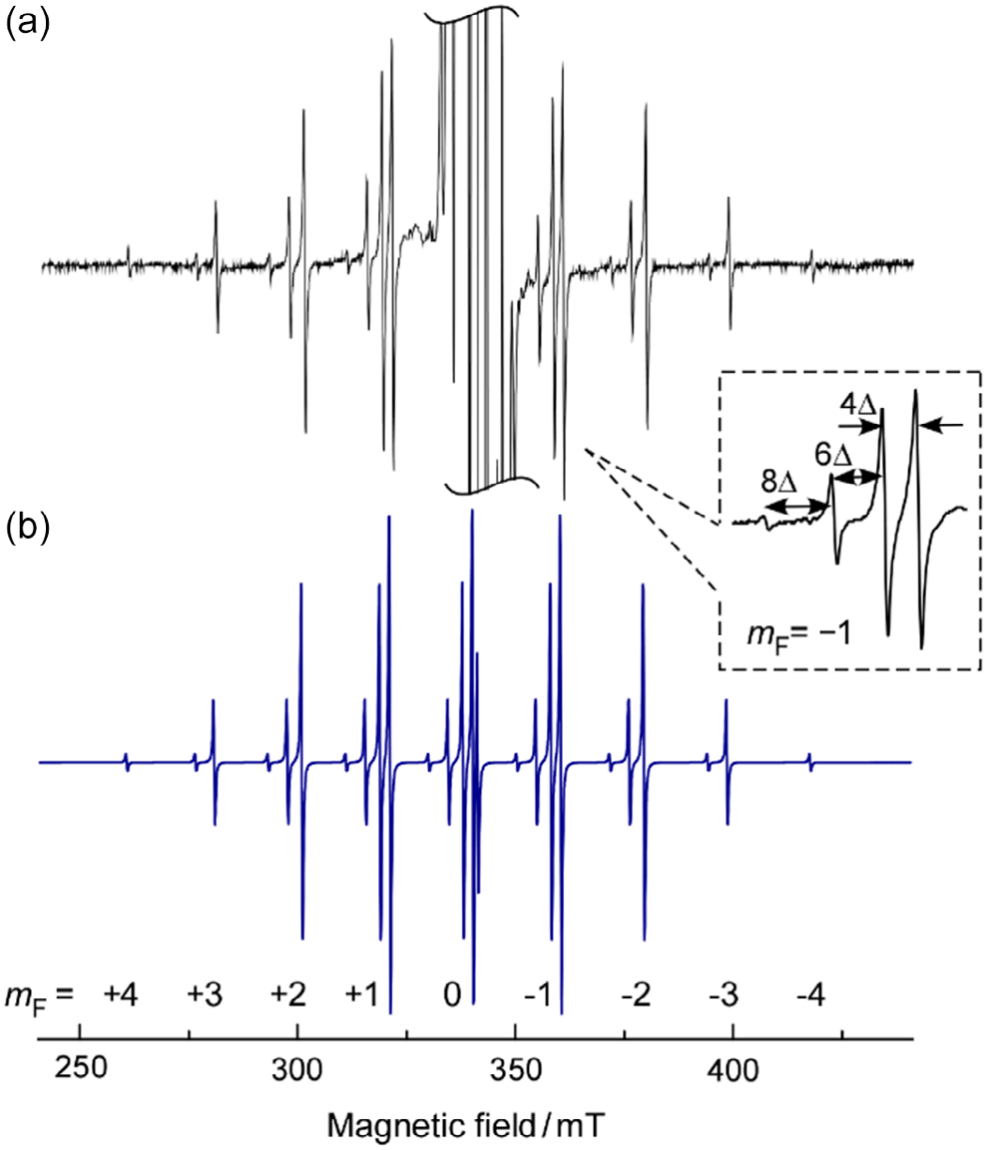
(a) EPR spectrum at 77 K for an HME matrix containing 1 mol% C_8_F_8_, recorded after *γ*‐ray irradiation with a absorbed dose to 10 kGy at 77 K and subsequent annealing at 140 K. (b) Simulated EPR line shape fitted to (a), incorporating higher‐order effects. *m*_F_: spin magnetic quantum numbers for each of the total nuclear spin quantum number, 4, 3, 2, 1, 0. Inset: expanded view of the signal band at *m*_F_ = −1. Reproduced with permission from ref. [69]. Copyright 2022, AAAS.

Figure 12a shows the EPR spectrum of C_8_F_8_^−^ measured at 77 K after *γ*‐ray irradiation (10 kGy, ^60^Co‐radiation facility, Faculty of Engineering, HiroshimaUniversity) of hexamethylethane (HME) as a matrix containing ≈1 mol% of C_8_F_8_. Although a strong central signal originating from the matrix was observed, nine absorption bands with a hf splittings of ≈19 mT appeared upon vertical magnification of the spectrum, as shown in the figure.

Among the nine absorption bands, the central oneoverlaps with the strong signal originating from the matrix and therefore could not be clearly identified. The spacing between the bands, ≈19 mT, corresponds to an unusually large hf splitting that cannot be explained by typical organic radicals. This value is most reasonably attributed to fluorine nuclei, as no other nuclei are consistent with such a large hf splitting (^19^F: nuclear spin 1/2, natural abundance 100%). From the nine absorption bands, it is suggested that eight fluorine atoms are interacting with the unpaired electron, and that those fluorine atoms are completely equivalent. Based on this observation, the spectrum shown in Figure 12a is attributed to the radical anion of perfluorocubane (C_8_F_8_^−^).

The isotropic hf splitting of the fluorine atoms, *a*(^19^F), calculated by DFT at the B3LYP/6‐311++G(d, p) level, was 19.24 mT, which is in excellent agreement with the experimental value of 19.62 mT [69]. Based on this the *a*(^19^F) value, the spin density of the unpaired electron distributed over the 2 s orbitals of the eight fluorine atoms was estimated to be ≈9% in total.

When the spin density is equal on the 1s orbital of hydrogen and the 2 s orbital of fluorine, the isotropic hf splitting of fluorine becomes over 30 times larger than that of hydrogen [1, 2, 70]. Such large hf splitting values cause second‐order energy shifts exceeding the linewidth, resulting in observable peak splitting. This is identical to the explanation previously given for the high‐resolution spectrum of the methyl radical. In the EPR spectrum of C_8_F_8_^−^, all but the outermost peaks (*m*_F_ = ±4) are split into multiple components due to these effects, as described by the following equation [1, 2, 71].



(9)

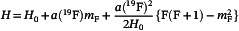

where *H* is the magnetic field at which the peak appears, *H*_0_ is the center field of the spectrum, F is the total nuclear spin, and *m*_F_ is the magnetic quantum number of each nucleus. By defining the peak shift width as Δ = *a*(^19^F)^2^/(2*H*_0_), a calculation using the actual values yields Δ = 0.565 mT. Using the value of Δ, no indication of the Δ shift is provided in the figure, but the peak corresponding to *F* = 1 is shifted by the Δ value toward the lower magnetic field side from its position predicted by the high‐field approximation. Similarly, the peaks for *F* = 2, 3, and 4 are shifted by 5Δ, 11Δ, and 19Δ, respectively (Figure 12). The intensity of each peak reflects the density of states for the spin magnetic quantum number; for example, the four peaks corresponding to *m*_F_ = +1 exhibit relative intensities of 1:7:20:28 from the low‐field side. The simulated EPR line shape, which incorporates higher‐order perturbation effects, is shown inFigure 12b, and it agrees very well with the experimental spectrum. The simulation was performed using dedicated software (WINEPR SimFonia, ver.1.25, Bruker) with the following isotropic EPR parameters: *g*‐value = 1.9985, *a*(^19^F) = 19.62 mT (eight F atoms), and linewidth = 0.35 mT [69]. In the HME matrix, despite being in a solid state at the lowtemperature of 77 K, the EPR spectrum of the C_8_F_8_^−^ exhibits an isotropic line shape, as if it were in a liquid phase. This observation indicates that the C_8_F_8_^−^ radical anion molecules undergo random rotational motion without translational movement of its center of mass, a behavior previously reported in several matrices including HME [13, 26, 72, 73, 74]. While the disappearance of anisotropy in hf structures and *g*‐values simplifies the spectral features and facilitates analysis, it also presents a drawback in that information regarding the static electronic structure.

DFT calculations (B3LYP/6‐311++G(d, p)) reveal that the SOMO of the C_8_F_8_ radical anion closely resembles that of the neutral species as shown in Figure 11b, where relatively high electron density regions are exhibited (isovalue = 0.06, based on a unit system where a value of one corresponds to one electron in a cube with 0.1 nm sides) [68]. When the isovalue threshold is lowered, it becomes apparent that the molecular orbital with opposite phase to the interior of the cage extends along the C—C bonds and envelops the outer surface of the cubane framework. Nevertheless, it is evident that a significant portion of the unpaired electron is localized within the cubane skeleton. This unusual electronic structure is particularly intriguing, as it appears to play a crucial role in stabilizing the C_8_F_8_ radical anion.

In future studies, MI‐EPR measurements using different types of matrices are expected to provide new insights into the electronic structure of C_8_F_8_^−^, such as the anisotropic hf splittings of fluorine atoms and substituent effects. With its simple cubic carbon framework and the ability to trap an electron within, C_8_F_8_ is a uniquely attractive molecule. Further studies on its electron‐accepting properties, aggregation behavior, and potential applications are highly anticipated.

## 5 Summary and Outlook

EPR spectroscopy provides detailed information not only on radical identification and quantification, but also on their geometric and electronic structures and reaction dynamics. When combined with MI and ionizing radiation (e.g., *γ*‐rays, X‐rays), EPR enables the characterization of transient radical intermediates—including cationic, anionic, and neutral species—in low‐temperature solids.

Recent advances in computational methods, particularly DFT, have significantly improved the accuracy of EPR parameter predictions, such as hf splittings, for both neutral and charged radicals. This personal account highlights EPR studies conducted by the authors, including quantum dynamics and super‐hf analysis of methyl radicals, H···CH_3_ triplet pairs, and H···H_2_ complexes in argon matrices. It also presents structural distortions due to second‐order Jahn–Teller effects in silacyclohexane radical cations in halocarbon matrices, and EPR characterization of the newly synthesized perfluorocubane radical anion using TME matrices.

MI‐EPR is a powerful technique for probing unstable paramagnetic species at the molecular level and plays a vital role in molecular science. Its potential as a laboratory model for studying chemical evolution in space is increasingly recognized, and further developments in this field are highly anticipated.

## Conflicts of Interest

The author declares no conflicts of interest.

## Data Availability

The data that support the findings of this study are available from the corresponding author upon reasonable request.
